# Coherence of Influenza Surveillance Data across Different Sources and Age Groups, Beijing, China, 2008-2015

**DOI:** 10.1371/journal.pone.0169199

**Published:** 2016-12-30

**Authors:** Zhenyu Wu, Xiaoyu Sun, Yanhui Chu, Jingyi Sun, Guoyou Qin, Lin Yang, Jingning Qin, Zheng Xiao, Jian Ren, Di Qin, Xiling Wang, Xueying Zheng

**Affiliations:** 1 Department of Biostatistics, School of Public Health, Key Laboratory of Public Health Safety and Collaborative Innovation Center of Social Risks Governance in Health, Fudan University, Shanghai, China; 2 Xicheng District Centers for Disease Control and Prevention, Beijing, China; 3 School of Nursing, The Hong Kong Polytechnic University, Hong Kong SAR, China; Columbia University, UNITED STATES

## Abstract

Influenza is active during the winter and spring in the city of Beijing, which has a typical temperate climate with four clear distinct seasons. The clinical and laboratory surveillance data for influenza have been used to construct critical indicators for influenza activities in the community, and previous studies have reported varying degrees of association between laboratory-confirmed influenza specimens and outpatient consultation rates of influenza-like illness in subtropical cities. However, few studies have reported on this issue for cities in temperate regions, especially in developing countries. Furthermore, the mechanism behind age-specific seasonal epidemics remains unresolved, although it has been widely discussed. We utilized a wavelet analysis method to monitor the coherence of weekly percentage of laboratory-confirmed influenza specimens with the weekly outpatient consultation rates of influenza-like illness in Beijing, China. We first examined the seasonal pattern of laboratory-confirmed cases of influenza A (subtyped into seasonal A(H1N1) and A(H3N2) and pandemic virus A(H1N1) pdm09) and influenza B separately within the period from 2008–2015; then, we detected the coherence of clinical and laboratory surveillance data in this district, specially examining weekly time series of age-specific epidemics of influenza-like illnesses in the whole study period for three age categories (age 0–5, 5–15 and 25–60). We found that influenza A and B were both active in winter but were not always seasonally synchronous in Beijing. Synchronization between age ranges was found in most epidemic peaks from 2008–2015. Our findings suggested that peaks of influenza-like illness in individuals aged 0–5 and 5–15 years consistently appeared ahead of those of adults, implying the possibility that schoolchildren may lead epidemic fluctuations.

## Introduction

In temperate developed regions, seasonal influenza has been well studied and be known as one of the main causes of substantial morbidity and mortality [1]. By identifying explicit and predictable annual seasonality, public health interventions can be implemented to prevent and control influenza. Investigating the seasonal patterns of influenza activity also allows for adequate preparations of preventive measures before the influenza season begins [1, 2]. It is usually assumed that the reported influenza activity is generally representative of the timing of influenza activity and that influenza virus infections can be identified in the laboratory. Nevertheless, the surveillance of influenza epidemics cannot only rely on increasing numbers of influenza-like illness (ILI) cases because non-specific ILI-like symptoms may be caused by etiologies other than influenza [3]. As a result, many countries collect data on laboratory-confirmed influenza infection parallel to clinical surveillance to provide more accurate and timely information about influenza virus activity than information from conducting clinical surveillance alone. Although there has been important progress in influenza surveillance systems in recent years, the volume of information on the surveillance of ILI and laboratory-confirmed virus activity remains too sparse for detailed analyses at the province and city levels even in developed countries [4, 5].

Many countries now include laboratory-confirmed influenza tests as part of their national influenza surveillance programs, and this information is communicated through the Internet to the public health community on a weekly basis [6–8]. In contrast, the surveillance in developing countries is largely falling behind due to the little data available on the surveillance of influenza activity in the regions. Because only a small proportion of influenza infections are confirmed through laboratory findings, empirical data on laboratory-confirmed seasonal influenza are limited by very low and possibly non-systematic case ascertainment. In general, little information is available in the literature regarding the association between clinical and laboratory surveillance data in developing regions. We intend to identify the epidemic activity in a temperate region of a developing country and explore whether it is recurrent and multi-phasic in seasonal patterns.

Studies on age-specific epidemic activity curves could provide key evidence on the mechanism of virus transmission and could facilitate the formulation of age-specific control measures [9, 10]. There is a growing body of evidence supporting that human mobility may drive the dispersal of influenza virus activity [11–13]. However, this hypothesis has not been supported consistently; for example, some studies [14–16] questioned the hypothesis that the children actually bring about influenza epidemic fluctuations. The objective of this study is to identify the age groups in which influenza activity first peaks in the community using our extensive surveillance of ILI cases over eight years and thereby fill a gap in the data available for temperate developing regions.

## Methods

### Data sources

ILI is defined as a fever (temperature of 38°C or greater), cough and/or a sore throat in the absence of a known cause other than influenza. Weekly district-wide ILI time series from 2008–2015 were obtained from Beijing Medical Institutions in Communicable Disease Surveillance and Early Warning System (collecting data through inquiry and diagnosis by doctors in each hospital) and were categorized into five age groups (0–5, 5–15, 15–25, 25–60 and greater than 60 years). Weekly laboratory-confirmed influenza infections for influenza A and B from January 2008 to December 2015 were obtained from Xicheng District Centers for Disease Control and Prevention in Beijing, China. Throat swab specimens were extracted from patients that had not taken any antiviral drugs and within three days of the onset of symptoms. The isolation and identification of influenza virus in Madin Darby Canine Kidney (MDCK) cells were conducted in the laboratory of Xicheng District Centers for Disease Control and Prevention. As the capital and the second largest city in China, Beijing has a humid continental climate, and the monthly daily temperature in January is -3.7°C, while in July it is 26.2°C. The core functional area of Beijing and Xicheng District are 92.39 and 50.53 square kilometers, and population in the areas are 2,203 and 1,298 thousand in 2016, respectively [17]. That is, the density of the area is about 25 thousand people per square kilometer. As a central district in Beijing, Xicheng District has ten Grade III Level A hospitals (top-class in China). It should be stressed that the daily children outpatient visits to Beijing Children's Hospital affiliated with Capital Medical University in the district, represent more than half of all of the daily children outpatient visits to hospitals in the entire city [17, 18]. Given the highly compacted population and the homogeneity of both climate and geography among all districts in Beijing [17, 19], we believe that our data can be representative of the entire Beijing population’s influenza activity.

### Ethical statement

The collection of data from laboratory-confirmed cases was part of a long-term public health surveillance effort by the Chinese Centers for Disease Control and Prevention. We utilized weekly aggregated data that did not include individual patient information.

### Wavelet analysis

We utilized wavelet analysis, involving the transformation of a data series with a wavelet, to determine the timing of epidemics. Employing time-localized waves as basis functions. This approach can detect non-stationary behavior, which changes over time in both frequency and amplitude. In the analysis of epidemiological time series data, wavelet analysis has been used previously to measure synchrony in influenza activity between two locations or between two incidence proxies [20]. We adopted a global wavelet spectrum to estimate the amplitude of annual and semi-annual epidemic cycles for subtypes of the weekly percentage of laboratory-confirmed influenza specimens. As a measure of epidemic coherence between laboratory surveillance data and the weekly percentage of clinical ILI consultation, as well as between different age groups, the cross wavelet transform method was employed and high coherence suggested that one time series was associated with the other one at a particular time and frequency [21]. We estimated the weekly phase angle difference in wavelet-reconstructed time series after extracting the main annual cycle (52-week period; Morlet continuous wavelet). The Morlet wavelet is essentially a wavelet consisted of a complex exponential, which can capture the cyclical fluctuations in local time series [22]. Wavelet analysis and coherence comparisons were implemented in R version 3.2.4 using the sowas package by [23, 24] and some functions written by [25] to estimate the amplitude of the annual and semi-annual epidemic cycles in each wave. Following Maraun et al. [23], we estimated the coherence by smoothing 3 periods in the time direction and one octave (0.5 in each direction) in scale direction. The following results were not sensitive by changing these parameters.

## Results

### Differences in weekly percentage of specimens positive for influenza with influenza A and B from 2008 to 2015

**Fig 1:**
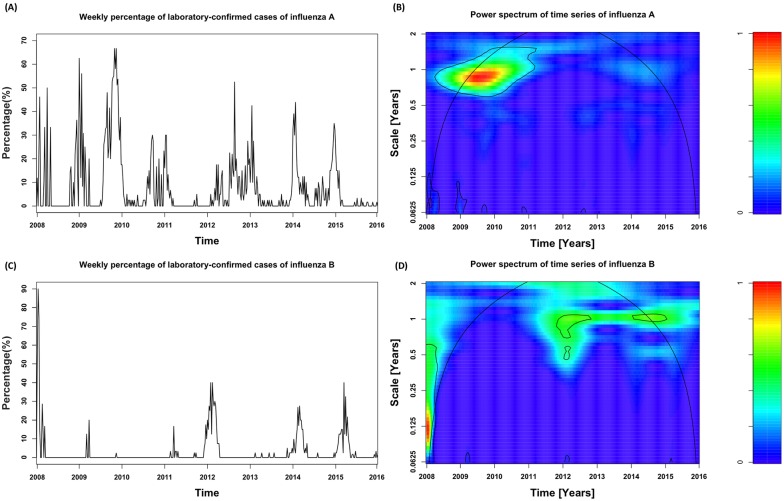
Wavelet analysis of weekly percentage of positive laboratory-confirmed cases of influenza A and B. (A) Weekly percentage of laboratory-confirmed cases of influenza A; (B) Power spectrum of time series of percentage of confirmed cases of influenza A; (C) Weekly percentage of laboratory-confirmed cases of influenza B; (D) Power spectrum of time series of percentage of confirmed cases of influenza B. The black solid contour lines indicate the regions of power significant at the 95% confidence level which can be assumed to be a true feature. The region outside the black-curved cone indicates the presence of edge effects and is not the evidence for conclusions. The power values were shown in the panel on the right.

Table 1 summarized the annual numbers of laboratory-confirmed influenza infections and ILI in the Xicheng District of Beijing from 2008–2015. Fig 1A showed the weekly percentage of laboratory-confirmed cases of influenza A. The mean was 7.8% and ranged between 0% to 67%. There were no clear epidemic periods after 2012, despite two sharp spikes around the winters of 2009 and 2010. Fig 1B showed the wavelet power spectrum of the weekly percentage of laboratory-confirmed positive specimens of influenza A. A high power indicates frequency- and time-specific periodicity. As shown in Fig 1B, although annual influenza epidemics were identifiable in most years (not colored as pure blue), the wavelet analysis revealed that the influenza activities for type A showed a statistically significant annual pattern with one peak in winter in the years 2009–2010. Fig 1C showed weekly percentage of laboratory-confirmed cases of influenza B. The mean was 2.9% and ranged from 0 to 90%. In Fig 1C, influenza B showed one peak in the winter in 2012, 2014 and 2015, which was consistent with the annual cycle detected in Fig 1D. Only an annual seasonal pattern was noticeable in Beijing.

**Table 1:** Annual numbers of specimens tested for influenza infection, number of positive specimens (including type A, type B and uncategorized), averaged weekly percentage (AWP) of influenza cases and averaged weekly consultation rates of influenza-like illness, 2008–2015.

Time	No. Laboratory	No. A	No. B	No. Positive	AWP	Rates ILI
2008	338	43	75	118	0.123	0.048
2009	1429	430	3	499	0.265	0.041
2010	1964	111	0	480	0.232	0.044
2011	1406	39	27	66	0.039	0.036
2012	2028	183	134	317	0.152	0.038
2013	2089	83	11	94	0.045	0.029
2014	2085	216	86	302	0.144	0.023
2015	2860	44	113	159	0.067	0.019
**Total**	14253	1149	449	2035	0.143	0.034

### Coherence in weekly percentage of specimens positive for influenza and percentage of ILI consultations from 2008 to 2015

**Fig 2:**
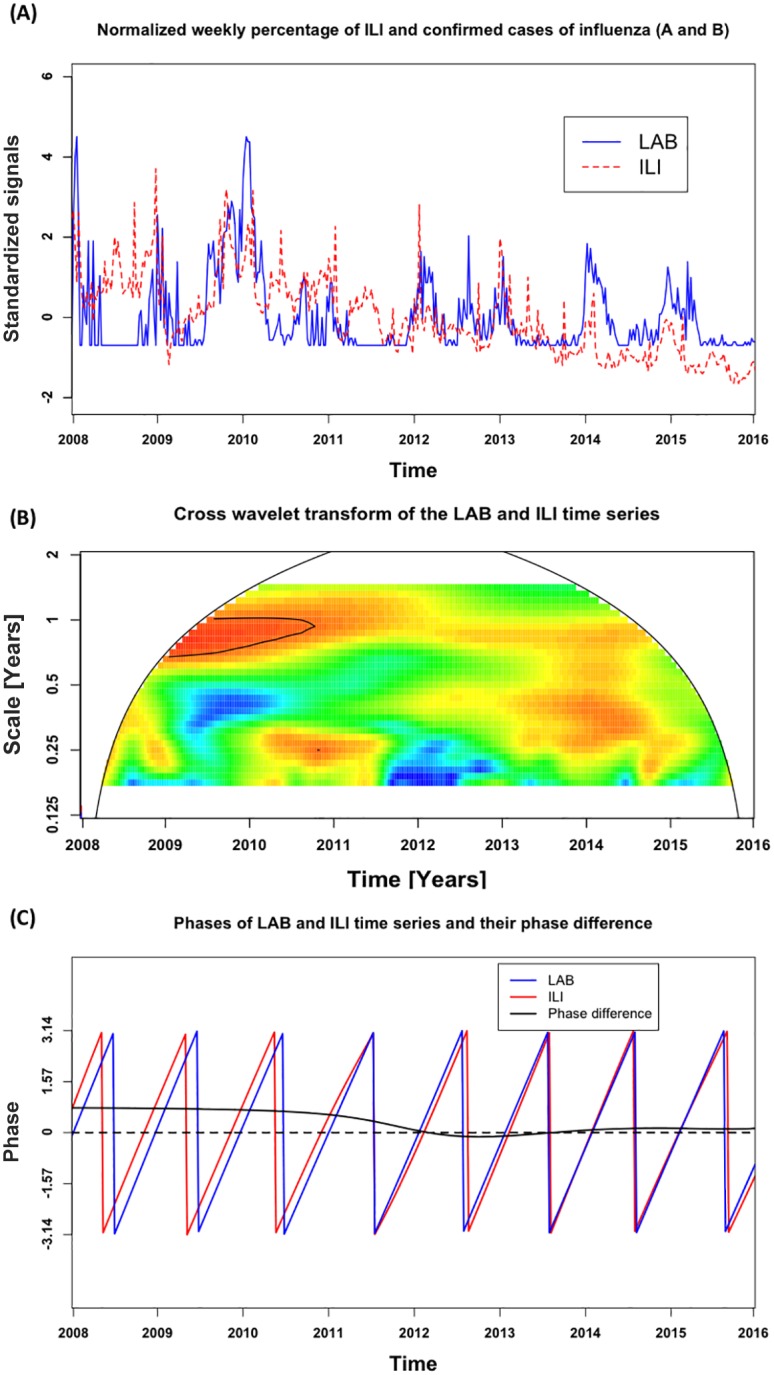
Association between normalized weekly percentages of ILI consultations (ILI) and weekly proportion of positive laboratory-confirmed cases of influenza A and B (LAB). (A) Normalized weekly percentage of ILI consultations and laboratory-confirmed cases of influenza (A and B); (B) Cross wavelet transform of the LAB and ILI time series. The power values were colored in Fig 2B. The black solid contour lines indicate the regions of power significant at the 95% confidence level which can be assumed to be a true feature. The region outside the black-curved cone indicates the presence of edge effects and is not drawn as the evidence for conclusions. (C) Phases of LAB and ILI time series (solid lines, colors as in Fig 2A) and their phase difference (black dashed lines).

The time series of weekly percentage of laboratory-confirmed influenza specimens (LAB) and weekly percentage of clinical consultations for ILI were normalized with mean and standard deviations as zero and one, respectively. After normalizing and aligning the curves of the weekly laboratory-confirmed rates and ILI consultation rates from 2008 to 2015, the irregular shapes of the epidemic curves for Xicheng District, Beijing, are illustrated in Fig 2A, showing both the level of synchronization as well as a similarity in shape. The results of wavelet coherence analysis between LAB and ILI are shown in Fig 2B. The LAB data presented a consistently significant coherence with ILI for the annual cycle during 2009–2011 and 2014–2015 on 95% significance level. For the semiannual cycle, high coherence was found only in the year 2014. To compare the timing of LAB and ILI, we calculated their phase angle difference for the annual cycle that was most clearly observed throughout the study. The mean of the phase angle difference was 0.328 in radians as shown in Fig 2C, which implied that LAB was estimated to follow ILI with an average delay of 2.716 weeks. ILI consistently preceded LAB from 2008 to early 2011 and approached the latter thereafter.

### Age-specific epidemic waves of influenza in Beijing

**Fig 3:**
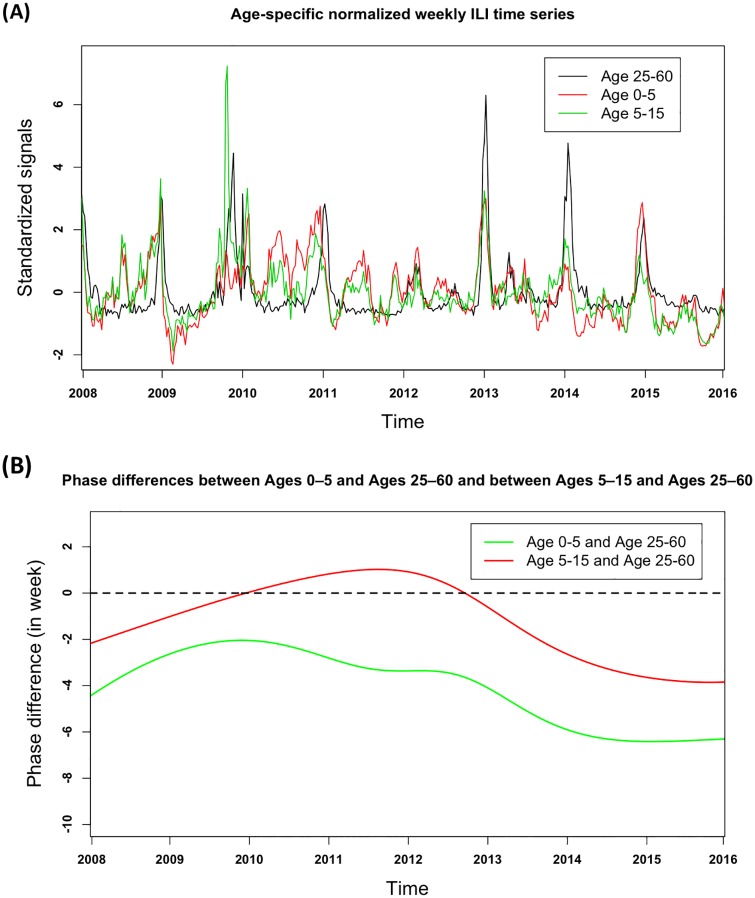
Association between weekly percentage of ILI consultations in the Age 0–5, 5–15 and Age 25–60 groups. (A) Age-specific normalized weekly ILI time series for Ages 0–5 (red), Ages 5–15 (green), and Ages 25–60 (black); (B) Phase differences between Ages 0–5 and Ages 25–60 (red dashed lines) and between Ages 5–15 and Ages 25–60 (green dashed lines).

In Fig 3A, we found that influenza virus activity was synchronized across age groups throughout the whole study period. The figure showed that consistent peak patterns were observed for the epidemic peak times of ILI among the three groups, especially for three obvious peaks in years 2013–2015. The composite epidemic pattern was also remarkably similar in the waves for the age groups 0–5, 5–15, and 25–60. One noticeable distinction between the curves of ages 0–5 and 5–15 and that of ages 25–60 during the pandemic in 2009–2011 was that the peak in the 5–15-year-old age group appeared much sharper than that of the adults of aged 25–60, and the influenza pandemic patterns appeared highly heterogeneous at each age scale. This phenomenon only appeared during the pandemic, but not in any other peak of the year, such as those in 2011 and 2013–2015. To compare the timing across various age groups, we calculated their phase angle difference for the annual cycle that was consistently observed throughout the study in Fig 3B. The 25–60 age group was found to follow the 0–5 group, with an average delay of 4.1 weeks, ranging from the delay of 2.0 to 6.4 weeks, and to follow the 5–15 group, with an average delay of 1.1 weeks, ranging from 1.0 week ahead to 3.9 weeks’ delay.

## Discussion

Influenza epidemics in temperate latitudes are usually characterized by the dominance of influenza B or one of two subtypes of influenza A, A/H3N2 or A/H1N1. Goldstein et al. [26] found that the epidemic sizes of influenza A/H3N2, A/H1N1, and B infections varied from year to year in temperate regions and discovered that type A was the most virulent of the three types of influenza virus and was associated with seasonal epidemics in temperate regions. Rambaut et al. [27] confirmed that the epidemiological data on influenza A demonstrated an inconsistent seasonal pattern of influenza virus infection across years, with high activity during the winter. As shown in Fig 1A, the shape of the epidemic curve in Beijing was irregular when compared to the typical epidemic curve described by epidemic models, which raised unique challenges. Unlike in most temperate regions [28, 29] the influenza circulation in Beijing presented a less well-defined seasonality when compared with developed countries. One possible reason for this finding is that our study started around the period of the influenza pandemic when the appearance of H1N1pdm09 interrupted the regular variations of influenza A and this might have lasted for several years. The laboratory-confirmed cases of influenza B, however, turned out to be seasonal in the post-pandemic period, possibly because they were less influenced by the pandemic.

Annual cycles were observed in both the laboratory-confirmed influenza and ILI activity data, and both disease indicators were highly synchronous for most of the study period. Peak timing provides a complementary measure of synchrony between influenza surveillance systems. Yang et al. [30] mentioned that although the weekly percentage of laboratory-confirmed influenza specimens appeared to be temporally representative of the level of influenza activity at the community level, this was not the case during the 2009 pandemic, when laboratory practices varied in response to the public health needs. During the pandemic period, clinical and laboratory testing procedures varied in response to public health and clinical needs and laboratory capacity, and thus the detection rates were not constant over this period. We showed that the percentages of ILI consultations were in synchrony with laboratory surveillance findings in the period of the pandemic and therefore can be adopted as a reliable indicator of influenza epidemics during special periods.

In our study, during both the pandemic period and peak timing from 2008–2015, laboratory surveillance and clinical surveillance data showed high retrospective correlation in capturing season-to-season epidemic timing and magnitude. However, we noticed that from 2008 to early 2011, these data sources were not exactly synchronous, and thus significant phase differences were captured by wavelet analysis. Yang et al. [30] stated that other common respiratory viruses had no obvious epidemic peaks and were delayed during the pandemic of A/H1N1 2009 pandemic. Additionally, competition and interference between influenza A viruses and other respiratory viruses existed. Therefore, we suspected that other viruses were active during the period, which could lead to clinical surveillance being less specific to influenza. Although laboratory data are considered to be more rigorous and accurate than clinical data, the surveillance system was constructed only since 2007 in China [5] and therefore limits the length of time series we can obtain so far. Further research is required to determine which time series can be trusted to demonstrate and predict influenza activity in Beijing.

Past work suggested there was essentially no lead between reference laboratory surveillance system and ILI cases in Hong Kong in 2009 [6]. Other published studies have indicated that ILI surveillance was usually 2–3 weeks ahead of laboratory surveillance in public health practices [2, 31]. Our findings regarding surveillance data in Beijing are consistent with these studies, and thus support that clinical ILI consultation rates could be a reliable and timely indicator of changes in influenza virus activities in Beijing, a city with temperate climate. The detection of the coherence between the confirmed influenza cases and ILI can be applied to other temperate regions to provide insights into the seasonality of influenza viruses in temperate regions. Meanwhile, laboratory confirmation will likely play an increasingly important role in the development of better methods of early detection and summary measures of influenza activity in key times. Our study provides certain evidence of the seasonality and periodicity of both surveillance and clinical data, such as the local features of a single time series and the local coherence between two time series. In practice, when ILI is consistently detected ahead of laboratory-confirmed cases, it can bring up an early warning for influenza epidemic, for susceptive populations to improve their self-protection, and for the government to allocate medical resources timely. These results will need to be verified by future research with access to greater number of laboratory-confirmed influenza cases. It is hoped that the size of laboratory samples can be increased to provide better evidence to facilitate the surveillance of both clinical and laboratory samples, both of which can be of great use in public influenza activity prediction.

When divided by age groups, clinically-diagnosed ILI indicators accurately capture weekly fluctuations in influenza activity during inter-pandemic and pandemic seasons and can be utilized to assess the effects of age on trends in epidemic activity. Using wavelet coherence analysis to compare these time series, we did not observe large differences in susceptibility from season to season. In the pandemic of 2009, the peak of the ILI wave for age 5–15 was extremely high for two possible reasons. The weak immune system of teenagers may not have been able to resist the substantial threats presented by new viruses. Additionally, schoolchildren had few ways to avoid cross-infections because classes were suspended for only a short period of time. The main limitation of the approach we used to study influenza epidemics is that we need to be cautious when interpreting distinctions because it is hard to determine whether it was the epidemic activities among school children that drove the pandemic after 2009 or whether the pandemic itself acted as a major destructive effect on school children in the following two years. Slight but consistent age-specific differences became apparent after identifying phase difference. We found that the peaks of influenza-like illness in the time series of groups aged 0–5 and 5–15 consistently appeared ahead of those of adults, implying the possibility that schoolchildren may lead epidemic fluctuations of clinical ILI consultations. Further research is needed to confirm this subtle difference. Beyond the analysis presented here, it is plausible that a thorough understanding of age-specific driving forces of seasonal epidemics will require additional long-term data from other temperate regions.

## Supporting Information

S1 Data Weekly data of Xicheng District, Beijing, China, 2008–2015. (XLS)
